# Impact of Ammonium on Syntrophic Organohalide-Respiring and Fermenting Microbial Communities

**DOI:** 10.1128/mSphere.00053-16

**Published:** 2016-04-20

**Authors:** Anca G. Delgado, Devyn Fajardo-Williams, Kylie L. Kegerreis, Prathap Parameswaran, Rosa Krajmalnik-Brown

**Affiliations:** a Swette Center for Environmental Biotechnology, Biodesign Institute, Arizona State University, Tempe, Arizona, USA; b School of Sustainable Engineering and the Built Environment, Arizona State University, Tempe, Arizona, USA; University of Michigan

**Keywords:** *Dehalococcoides mccartyi*, *Geobacter*, ammonia, fermentation, organohalide respiration, trichloroethene

## Abstract

Syntrophic interactions between organohalide-respiring and fermentative microorganisms are critical for effective bioremediation of halogenated compounds. This work investigated the effect of ammonium concentration (up to 4 g liter^−1^ NH_4_^+^-N) on trichloroethene-reducing *Dehalococcoides mccartyi* and *Geobacteraceae* in microbial communities fed lactate and methanol. We found that production of ethene by *D. mccartyi* occurred in mineral medium containing ≤2 g liter^−1^ NH_4_^+^-N and in landfill leachate. For the partial reduction of trichloroethene (TCE) to *cis*-dichloroethene (*cis*-DCE) at ≥1 g liter^−1^ NH_4_^+^-N, organohalide-respiring dynamics shifted from *D. mccartyi* and *Geobacteraceae* to mainly *D. mccartyi*. An increasing concentration of ammonium was coupled to lower metabolic rates, longer lag times, and lower gene abundances for all microbial processes studied. The methanol fermentation pathway to acetate and H_2_ was conserved, regardless of the ammonium concentration provided. However, lactate fermentation shifted from propionic to acetogenic at concentrations of ≥2 g liter^−1^ NH_4_^+^-N. Our study findings strongly support a tolerance of *D. mccartyi* to high ammonium concentrations, highlighting the feasibility of organohalide respiration in ammonium-contaminated subsurface environments.

**IMPORTANCE** Contamination with ammonium and chlorinated solvents has been reported in numerous subsurface environments, and these chemicals bring significant challenges for *in situ* bioremediation. *Dehalococcoides mccartyi* is able to reduce the chlorinated solvent trichloroethene to the nontoxic end product ethene. Fermentative bacteria are of central importance for organohalide respiration and bioremediation to provide *D. mccartyi* with H_2_, their electron donor, acetate, their carbon source, and other micronutrients. In this study, we found that high concentrations of ammonium negatively correlated with rates of trichloroethene reductive dehalogenation and fermentation. However, detoxification of trichloroethene to nontoxic ethene occurred even at ammonium concentrations typical of those found in animal waste (up to 2 g liter^−1^ NH_4_^+^-N). To date, hundreds of subsurface environments have been bioremediated through the unique metabolic capability of *D. mccartyi*. These findings extend our knowledge of *D. mccartyi* and provide insight for bioremediation of sites contaminated with chlorinated solvents and ammonium.

## Abstract (precis)

Contamination with ammonium and chlorinated solvents has been reported in numerous subsurface environments, and these chemicals bring significant challenges for *in situ* bioremediation. *Dehalococcoides mccartyi* is able to reduce the chlorinated solvent trichloroethene to the nontoxic end product ethene. Fermentative bacteria are of central importance for organohalide respiration and bioremediation to provide *D. mccartyi* with H_2_, their electron donor, acetate, their carbon source, and other micronutrients. In this study, we found that high concentrations of ammonium negatively correlated with rates of trichloroethene reductive dehalogenation and fermentation. However, detoxification of trichloroethene to nontoxic ethene occurred even at ammonium concentrations typical of those found in animal waste (up to 2 g liter^−1^ NH_4_^+^-N). To date, hundreds of subsurface environments have been bioremediated through the unique metabolic capability of *D. mccartyi*. These findings extend our knowledge of *D. mccartyi* and provide insight for bioremediation of sites contaminated with chlorinated solvents and ammonium.

## INTRODUCTION

The organohalide-respiring bacterium *Dehalococcoides mccartyi* ultimately catalyzes the reduction of the chlorinated solvents perchloroethene (PCE) and trichloroethene (TCE) to nontoxic ethene through *cis*-dichloroethene (*cis*-DCE) and vinyl chloride (VC) (1, 2). Bioremediation of subsurface environments using *D. mccartyi* has been an invaluable treatment technology, with hundreds of applications at contaminated sites (3). A significant challenge for *in situ* bioremediation arises when chlorinated ethenes are present in mixtures with other pollutants. PCE and TCE cooccur with other halogenated organic solvents (4). The presence of halogenated organics has been shown to impede chlorinated ethene reductive dehalogenation (5–8). Nitrogen (N)-containing compounds are also cocontaminants in PCE- and TCE-impacted groundwater, land, and landfills (9–12). Contamination with ammonium-N stems from numerous sources, including sewage and water main leakage, septic tanks, industrial spillages, river or channel infiltration, fertilizers, agricultural runoff, and landfill leachate (9–11). (In this report, the term “ammonium” comprises NH_4_^+^ and NH_3_ species; where appropriate, the chemical formulas are used to distinguish the species.) To date, 135 U.S. National Priorities List hazardous waste sites (compiled by the Agency for Toxic Substances and Disease Registry of the U.S. Centers for Disease Control and Prevention) are polluted with high concentrations of ammonium-N (13).

In the subsurface, *D. mccartyi* coexists alongside other terminal electron acceptor-respiring, fermenting, acetogenic, and homoacetogenic bacteria and also methanogenic archaea (14–18). Organohalide respiration and its syntrophic or competing microbial processes are usually studied in enrichment cultures derived from groundwater, soil, or sediment (see Table 1 in Delgado et al. [14]). These syntrophic, more simplified microbial communities containing *D. mccartyi* are also utilized for bioaugmentation applications at contaminated sites (3). Fermentative bacteria are of central importance for organohalide respiration to provide *D. mccartyi* with H_2_, their electron donor, acetate, their carbon source (2), specific amino acids (19), and vitamin B_12_ (20), and to alleviate CO toxicity (21). While *D. mccartyi* is a prerequisite for obtaining reductive dehalogenation to ethene, its mere presence in an environment does not ensure this outcome (3, 14, 22). It is well recognized that the success of *in situ* bioremediation is in part dependent on the composition and activity of the microbial community (23). Hence, unfavorable environmental conditions, toxicity, or inhibition impact directly (e.g., organohalide-respiring populations) or indirectly (e.g., fermentative or acetogenic bacteria) the transformation of chlorinated ethenes.

Ammonium is the preferred N source for growth of *D. mccartyi* (24) and is commonly provided as NH_4_Cl in the growth medium (5.6 mM or 0.08 g liter^−1^ NH_4_^+^-N). At high concentrations, however, ammonium generally exerts inhibitory effects on microbial activity (25, 26). Ammonia (NH_3_) readily diffuses into cells, where it becomes protonated, forming ammonium (NH_4_^+^) (27). Depletion of H^+^ from conversion of NH_3_ to NH_4_^+^ disrupts the proton motive force and energy acquisition required for growth (27–29) and can increase the intracellular pH and alter the cell redox potential (28). Persistence of ammonium-N is expected in the anoxic zones of groundwater where PCE and TCE are found. Typical ammonium-N concentrations in groundwater are in the milligram per liter range, whereas landfill leachates and animal waste stream concentrations are as high as 1 and 10 g liter^−1^, respectively (10, 30–33).

To date, studies delineating the effects of ammonium concentration on *D. mccartyi* and organohalide respiration in pure cultures or in mixed microbial communities have not been available. The key role of *D. mccartyi* in bioremediation demands a comprehensive understanding of the factors affecting syntrophic organohalide-respiring and fermenting microbial communities. Evidence from biohydrogen production has shown that some fermentative bacteria are able to resist inhibition to ammonium concentrations as high as 8 g liter^−1^ (25, 34, 35). However, the ammonium concentration contributed to lower rates of fermentation and longer lag times (34). In our study, we evaluated the effect of ammonium concentration on organohalide-respiring mixed microbial communities containing *D. mccartyi* and *Geobacteraceae* in batch experiments. We utilized quantitative tracking of products of TCE reductive dehalogenation, fermentation, homoacetogenesis, and methanogenesis in conjunction with the relative abundance of key genes within the microbial communities. We found that ammonium concentrations up to 2 g liter^−1^ ammonium-N did not impair ethene formation by *D. mccartyi* but significantly reduced dehalogenation and fermentation rates. Concentrations of ≥2 g liter^−1^ ammonium-N induced shifts in the lactate fermentation pathway from propionic to acetogenic. These findings underscore the importance of syntrophic microbial relations for organohalide respiration and extend our knowledge of *D. mccartyi*-containing communities in environments cocontaminated with chlorinated ethenes and ammonium.

## RESULTS AND DISCUSSION

**FIG 1:**
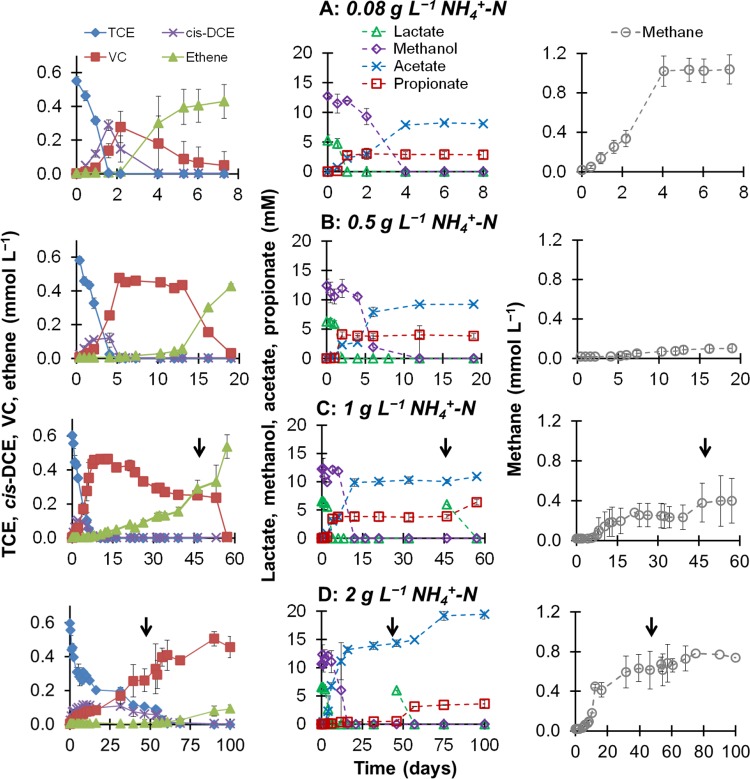
Reductive dehalogenation (left), fermentation (middle), and methanogenesis (right) in the presence of 0.08 (control) (A), 0.5 (B), 1 (C), or 2 g liter^−1^ NH_4_^+^-N (D). In panels C and D, the arrows accentuate the second addition of 6 mM lactate. The data are average results and standard deviations from triplicate cultures. The adjacent graphs are on the same time scale. Note the differences in the time scales between the graphs with different ammonium concentrations.

We evaluated the effect of ammonium concentration (expressed as NH_4_^+^-N) on TCE reductive dehalogenation in microbial communities fed the fermentable substrates lactate and methanol. In this report, the term “ammonium” comprises NH_4_^+^ and NH_3_ species; where appropriate, the chemical formulas are used to distinguish the species. Results of time course batch experiments in mineral medium containing up to 2 g liter^−1^ NH_4_^+^-N are presented in Fig. 1 (left). By days 5 and 8, 0.6 mmol liter^−1^ TCE was transformed to VC in the presence of 0.5 and 1 g liter^−1^ NH_4_^+^-N, respectively (Fig. 1B and C, left). Complete dehalogenation to ethene was achieved by day 19 at 0.5 g liter^−1^ NH_4_^+^-N (Fig. 1B, left), a concentration 6 times higher than in controls. VC, the dehalogenation product exclusively linked to *D. mccartyi* (2), was generated when ammonium was present at 2 g liter^−1^ NH_4_^+^-N (Fig. 1D, left) and also at 4 g liter^−1^ NH_4_^+^-N (see Fig. S1A and B in the supplemental material) within 100 days in the experiments. This activity is quite important given that these ammonium concentrations are typical of high-strength animal waste (30, 36, 37).

10.1128/mSphere.00053-16.2 Figure S1  Reductive dehalogenation and methanogenesis in the presence of 4 g liter^−1^ NH_4_^+^-N by ZARA-10 (A) or DehaloR^2 (B) microbial inocula. The cultures initially received 6 mM lactate and 12 mM methanol as electron donors and carbon sources (as for Fig. 1). In DehaloR^2 controls (0.08 g liter^−1^ NH_4_^+^-N), net production of methane was <0.14 mmol liter ^−1^ (data not shown). The data are average results with standard deviations from triplicate cultures. Download Figure S1, DOCX file, 0.1 MB. Copyright © 2016 Delgado et al. 2016 Delgado et al. This content is distributed under the terms of the Creative Commons Attribution 4.0 International license.

**FIG 2:**
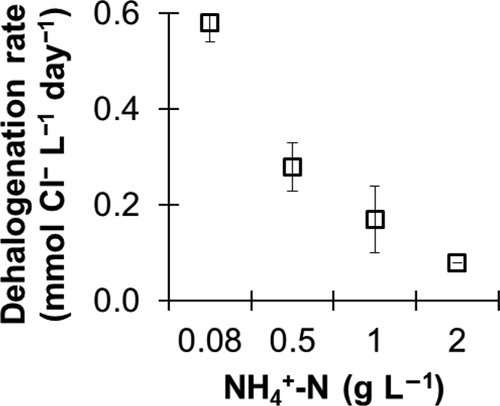
Effect of ammonium concentration on the maximum rate of reductive dehalogenation. The maximum rates were determined between two consecutive sampling points. The data are average results and standard deviations from triplicate cultures. The negative correlations between ammonium concentration and rate of dehalogenation were statistically significant at the α = 0.01 level (2 tailed; *n* = 12; Pearson correlation coefficient, *r* = −0.860; Spearman correlation coefficient, *ρ* = −0.972).

The maximum rates of dehalogenation observed in all cultures are shown in Fig. 2. The rates were negatively correlated with increasing ammonium concentration (Fig. 2). The correlation was determined to be statistically significant (Pearson *r* = −0.860; Spearman *ρ* = −0.972; α = 0.01 confidence level). The most prominent inhibitory effect was seen at 2 g liter^−1^ NH_4_^+^-N, where the rates of dehalogenation were 7 times lower than for controls (Fig. 2), resulting in prolonged time frames before generation of ethene.

**FIG 3:**
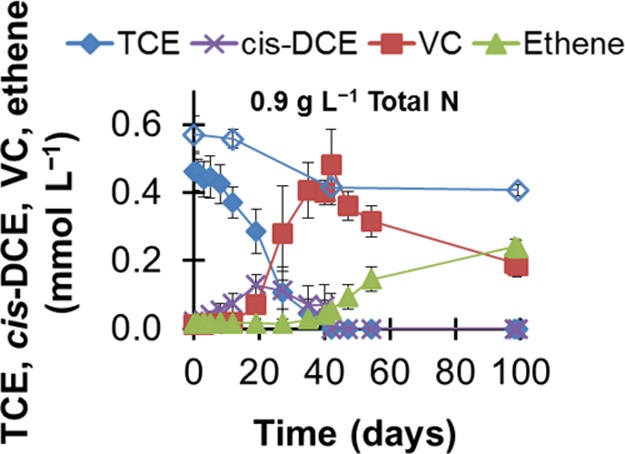
TCE reductive dehalogenation in anaerobic landfill leachate containing 0.6 g liter^−1^ NH_4_^+^-N and 0.9 g liter^−1^ total N. The empty diamonds are TCE concentrations in uninoculated controls. The data are average results and standard deviations from triplicate cultures.

Figure 3 compiles the dehalogenation activity in landfill leachate containing 0.6 g liter^−1^ NH_4_^+^-N and 0.9 g liter^−1^ total N. Landfills are prime examples of environments containing high ammonium levels and a variety of pollutants, including TCE and other chlorinated substances (31, 38–40). Our goal was to assess whether dehalogenation could be sustained in a landfill leachate sample with an ammonium concentration similar to what was tested in mineral medium experiments. In landfill leachate, by-products of TCE reduction were absent without the addition of the TCE-respiring microbial culture (Fig. 3) and with abiotic controls. In ZARA-10-inoculated leachate, ethene was the main product of TCE dehalogenation after 100 days (Fig. 3), albeit dehalogenation rates were lower than in defined mineral medium. The lower rates were expected and likely a consequence of the absence of added minerals, nutrients, and vitamins and the presence of cocontaminants and other electron acceptors in the landfill leachate.

We investigated closely the fate of the fermentable substrates, lactate and methanol, to determine the effects of ammonium concentration on fermentation pathways and to establish correlations between fermentation and reductive dehalogenation. The concentrations of organic fatty acids (lactic, acetic, and propionic) and methanol are shown in Fig. 1 (middle). Lactate was more rapidly depleted than methanol under all experimental conditions. Increasing the ammonium concentration prolonged the lag time before the onset of fermentation and lowered the fermentation rates for both substrates. In controls, lactate became nondetectable by day 1, while methanol was not consumed during this time (Fig. 1A, middle). The successive fermentations allowed us to determine the stoichiometry of the two substrates. Based on the measurements from Fig. 1 (middle), 0.61 ± 0.09 mM propionate and 0.43 ± 0.07 mM acetate were produced from 1 mol of lactate. Thus, lactate-fermenting bacteria were following the stoichiometry shown in equation 1 (Δ*G*°′ = −4.58 kJ/e− equiv):

(1) $$3\text{ C}{\text{H}}_{3}\text{C}{\text{H}}_{2}\text{OCO}{\text{O}}^{-}\to 2\text{C}{\text{H}}_{3}\text{C}{\text{H}}_{2}\text{CO}{\text{O}}^{-}+\text{C}{\text{H}}_{3}\text{CO}{\text{O}}^{-}+\text{HC}{\text{O}}_{3}{}^{-}+{\text{H}}^{+}$$

Lactate fermentation through this stoichiometry occurred not only in controls (0.08 g liter^−1^ NH_4_^+^-N) but also at 0.5 and 1 g liter^−1^ NH_4_^+^-N. In fact, at 0.5 and 1 g liter^−1^ NH_4_^+^-N, addition of excess ammonium allowed us to confirm and better examine this stoichiometric pathway due to the lower fermentation rates and an obvious plateau in acetate production on days 2 to 4 and 4 to 6, respectively (Fig. 1B and C, middle). Lactate fermentation in mixed organohalide-respiring communities has also been described to follow equation 2 (Δ*G*°′ = −0.33 kJ/e− equiv), with acetate and H_2_ as fermentation products (41–44):

(2) $$\text{C}{\text{H}}_{3}\text{C}{\text{H}}_{2}\text{OCO}{\text{O}}^{-}+2\;{\text{H}}_{2}\text{O}\to \text{C}{\text{H}}_{3}\text{CO}{\text{O}}^{-}+\text{HC}{\text{O}}_{3}{}^{-}+2\;{\text{H}}_{2}+{\text{H}}^{+}$$

While feasible, the thermodynamics of equation 2 clearly show that fermentation to acetate and H_2_ is less favorable. This is consistent with the observations from our study (Fig. 1A to C, middle) and past studies on organohalide-respiring and fermenting cultures (44–46). At 2 g liter^−1^ NH_4_^+^-N (Fig. 1D, middle), on the other hand, a striking result occurred for lactate fermentation. Addition of ammonium at this concentration led to a shift in the lactate fermentation pathway from that defined by equation 1 (propionic fermentation) to that of equation 2 (acetogenic fermentation). The net increase in propionate at 2 g liter^−1^ NH_4_^+^-N was 0.4 mM, compared to 3.54 ± 0.64 mM at 0.08, 0.5, and 1 g liter^−1^ NH_4_^+^-N. Additional testing described in Text S1 and illustrated in Fig. S1 and S2 in the supplemental material confirmed that this ammonium-induced pathway summarized in equation 2 is conserved at concentrations of ≥2 g liter^−1^ NH_4_^+^-N. When 6 mM lactate was supplemented for a second time in the cultures with 2 g liter^−1^ NH_4_^+^-N, a net production of 3.12 mM propionate was detected (Fig. 1D). Ammonium was measured in order to rule out the possibility that the recovery of lactate fermentation activity was not due to a decrease in ammonium concentration potentially from microbial ammonium oxidation. Initial (2,003 ± 6 mg liter^−1^) and final (1,965 ± 35 mg liter^−1^) concentrations confirmed no substantial ammonium consumption in these microbial communities.

10.1128/mSphere.00053-16.1 Text S1  Summary of the effect of 4 g liter^−1^ NH_4_^+^-N on reductive dehalogenation, methanogenesis, and fermentation. Download Text S1, DOCX file, 0.02 MB. Copyright © 2016 Delgado et al. 2016 Delgado et al. This content is distributed under the terms of the Creative Commons Attribution 4.0 International license.

10.1128/mSphere.00053-16.3 Figure S2  Net production of acetate and propionate from fermentation of lactate and methanol by ZARA-10 and DehaloR^2 cultures amended with 4 g liter^−1^ NH_4_^+^-N. Lactate at 6 mM and 12 mM methanol were added at time zero. Propionate concentrations produced were 0.30 mM and 0.04 mM by the ZARA-10 and DehaloR^2 cultures, respectively. The data are average results with standard deviations from triplicate cultures. Download Figure S2, DOCX file, 0.02 MB. Copyright © 2016 Delgado et al. 2016 Delgado et al. This content is distributed under the terms of the Creative Commons Attribution 4.0 International license.

Regardless of the initial ammonium concentration, considerable decreases in methanol concentrations occurred only after lactate was completely consumed (Fig. 1A to D, middle). For this reason, we were able to clearly separate acetate produced from lactate and acetate generated from methanol. Based on the measurements from Fig. 1 (middle), the observed bacterial methanol fermentation stoichiometry is shown in equation 3 (Δ*G*°′ = −3.11 kJ/e− equiv):

(3) $$2\;\;\;\text{C}{\text{H}}_{3}\text{OH}\to \text{C}{\text{H}}_{3}\text{CO}{\text{O}}^{-}+2\;\;\;{\text{H}}_{2}$$

H_2_ was measured using a gas chromatography-thermal conductivity detector (GC-TCD) system; however, H_2_ did not accumulate to detectable levels during the experiments, indicating concomitant production and consumption. Consistent between the cultures with increasing ammonium concentrations, 75% ± 0.06% of the electron equivalents from methanol were channeled toward acetate production (this distribution was also reported in PCE-respiring fill-and-draw bioreactors fed with methanol [47]). In our work, addition of lactate at 0.08 to 1 g liter^−1^ NH_4_^+^-N led to limited amounts of H_2_ when lactate and methanol were fed concomitantly. A careful examination of fermentation and TCE reductive dehalogenation revealed that dehalogenation was mostly associated with methanol fermentation (see Fig. S3 in the supplemental material).

10.1128/mSphere.00053-16.4 Figure S3  Fermentation of lactate and methanol, release of Cl^−^ from reductive dehalogenation, and methane production by ZARA-10 cultures in control experiments. This figure is a magnification of the results after the first 4 days (shown in Fig. 1A, top panels). Increases in Cl^−^ released and methane are mostly coupled to methanol fermentation. The data are average results with standard deviations for triplicate cultures. Download Figure S3, DOCX file, 0.04 MB. Copyright © 2016 Delgado et al. 2016 Delgado et al. This content is distributed under the terms of the Creative Commons Attribution 4.0 International license.

Anaerobic digestion studies have documented that methanogens display a higher sensitivity to high concentrations of ammonium than fermenters and acetogens (29, 48). Furthermore, methanogenic activity decreases with increasing ammonium concentration (37, 49, 50). Methanogenesis exhibited the highest activity in our control study (Fig. 1A, right). At 0.5, 1, and 2 g liter^−1^ NH_4_^+^-N, total methane production was diminished by 90%, 63%, and 41%, respectively, compared to controls. However, at 4 g liter^−1^ NH_4_^+^-N, methane concentrations similar to those in controls were observed after 46 days of incubation (see Fig. S1A in the supplemental material). Methane production was mostly coupled to methanol fermentation, as was reductive dehalogenation, and reached a plateau by day 4 in the controls (see Fig. S3 for better resolution of these reactions). The cultures containing 0.5 to 2 g liter^−1^ NH_4_^+^-N exhibited a lag time of 10 days or longer before methane production was detected (Fig. 1B to D, right). Interestingly, the trends in methanogenesis under excess ammonium conditions (≥0.5 g liter^−1^ NH_4_^+^-N) revealed an increase in methane produced as a function of increasing ammonium concentration (Fig. 1B to D, right; see also Fig. S1A). Methanogens have been shown to acclimate to ammonium concentrations as high as 3.5 g N liter^−1^ (49, 51), which concurs with the findings from our study for 2 to 4 g liter^−1^ NH_4_^+^-N.

**FIG 4:**
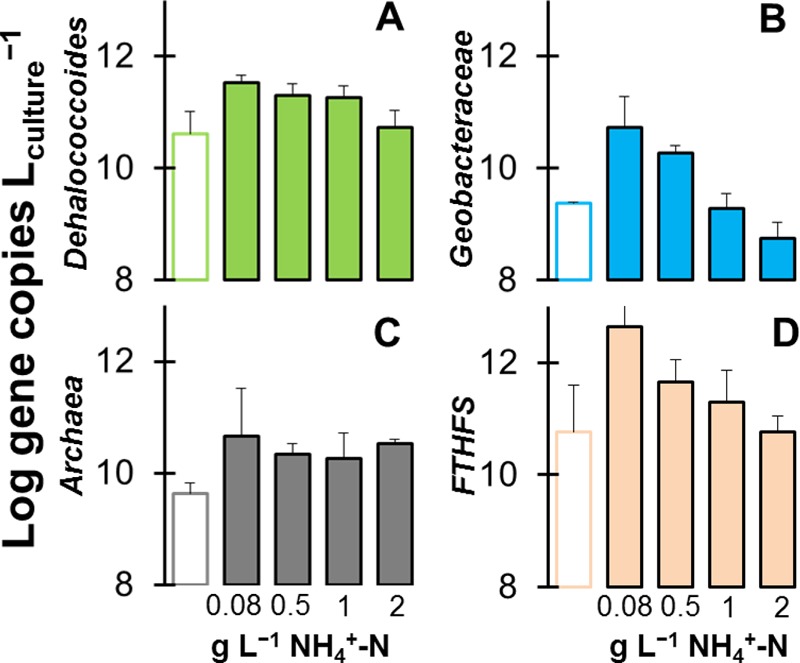
Quantitative PCR enumerating the 16S rRNA gene copies of *Dehalococcoides mccartyi*, *Geobacteraceae*, methanogenic *Archaea*, and homoacetogenic bacteria (FTHFS gene). The empty bars are the gene abundance levels at time zero. The filled bars are the log gene copy numbers at the end of the experiments: 0.08 g liter^−1^ NH_4_^+^-N (control) at day 8; 0.5 g liter^−1^ NH_4_^+^-N at day 19; 1 g liter^−1^ NH_4_^+^-N at day 60; 2 g liter^−1^ NH_4_^+^-N at day 100. The data are average results with standard deviations from triplicate cultures.

Chemical analyses clearly unveiled an effect of ammonium concentration on reductive dehalogenation, fermentation, and methanogenesis. Ammonium-induced changes were also reflected in the relative abundance of key microbial community members, as measured by quantitative PCR (qPCR) (Fig. 4). Growth of *D. mccartyi* and *Geobacteraceae*, methanogenic *Archaea*, and homoacetogens (which possess formyltetrahydrofolate synthase [FTHFS]) was highest in controls (Fig. 4). This was expected and in agreement with ammonium noninhibitory conditions. The gene abundances of *D. mccartyi* were lower when excess ammonium was provided (Fig. 4A). However, differences in *D. mccartyi* gene concentrations between cultures with 2 g liter^−1^ NH_4_^+^-N and the other conditions also reflected incomplete consumption of TCE to ethene within the experimental time frame. Remarkably, *Geobacteraceae* showed no growth relative to time zero at 1 or 2 g liter^−1^ NH_4_^+^-N. These data indicate that concentrations of ≥1 g liter^−1^ NH_4_^+^-N are highly inhibitory for *Geobacteraceae* and strongly suggest that *D. mccartyi* populations are the main TCE–to–*cis*-DCE organohalide respirers at these high ammonium concentrations.

The concentrations of archaeal 16S rRNA genes, predominated by those of hydrogenotrophic methanogens, mirrored closely the total methane production data shown in Fig. 1. In particular, at 2 g liter^−1^ NH_4_^+^-N, the archaeal gene copies and methane concentrations were highest when ammonium was present at inhibitory concentrations (but still lower than in controls). Homoacetogens, assayed based on the gene for FTHFS, decreased as a function of ammonium concentration (Fig. 4D). The interplay between hydrogenotrophic methanogens, homoacetogens, and organohalide-respiring *D. mccartyi* cells has been previously documented (52). It is possible that inhibitory ammonium concentrations (≥2 g liter^−1^ NH_4_^+^-N in our study) allow more H_2_ to be channeled toward methanogenesis, to the detriment of homoacetogenesis.

While ammonium and chlorinated solvent contamination has been reported in numerous environments (12, 38), research on organohalide metabolism in the presence of ammonium is lacking. In cases where groundwater is nitrogen limited, a source of ammonium is often added as a biostimulant to promote bioremediation or to overcome a stall in reductive dehalogenation. The findings from our study provide evidence for the effect of elevated ammonium concentrations on TCE organohalide respiration by *D. mccartyi* and *Geobacteraceae* in fermentative syntrophic microbial communities. Chemical analyses showed conserved metabolic functions (production of ethene from TCE) for organohalide respiration in the presence of up to 2 g liter^−1^ NH_4_^+^-N. However, molecular biological analyses support a change in organohalide-respiring population dynamics from *D. mccartyi* and *Geobacteraceae* to mainly *D. mccartyi* for the partial reduction of TCE to *cis*-DCE. Increasing the concentration of ammonium was coupled to lower metabolic rates, longer lag times, and lower gene abundances for all microbial processes studied. Given the elevated free NH_3_ concentrations (up to 1.4 mM), these observations infer that energy for growth was diverted from respiration and fermentation to pumping out NH_4_^+^ from inside the cells to overcome toxicity. Overall, our study provides evidence on the feasibility of organohalide respiration of chlorinated ethenes in ammonium-contaminated environments while highlighting important kinetic and thermodynamic limitations to be considered for bioremediation applications.

## MATERIALS AND METHODS

### Experimental conditions.

Reductive dehalogenation batch experiments were performed using mineral medium and landfill leachate in 160-ml glass serum bottles. Reduced anaerobic mineral medium buffered with 30 mM HCO_3_^−^ (pH 7.4) and amended with vitamins was prepared as previously described (14, 53). NH_4_Cl was supplemented to obtain 0.08, 0.5, 1, and 2 g liter^−1^ NH_4_^+^-N (6 to 143 mM NH_4_Cl). At pH 7.4, free NH_3_ represented 0.1 to 1.4 mM of the total NH_3_/NH_4_^+^ concentration. NH_4_Cl was the only source of N in these experiments.

Landfill leachate was procured from the Northwest Regional Landfill, Surprise, AZ. The landfill had detectable levels of dichloroethenes, dichloroethanes, dichloropropanes, and VC (data provided by the landfill facility). The collected leachate had 0.6 ± 0.01 g liter^−1^ NH_4_^+^-N, 0.9 ± 0.02 g liter^−1^ total N, 4,300 ± 30 mg liter^−1^ chemical oxygen demand, 4,400 ± 110 mg liter^−1^ alkalinity as CaCO_3_, and a pH of 8.2. Before using it in the study, the leachate was sparged with N_2_ gas for 15 min to promote anaerobic conditions. HCO_3_^−^ at 5 mM was added as buffer, and the pH was adjusted to 7.5 by using a 2.25 M HCl solution.

At the beginning of the experiments, each batch bottle received 90 ml medium or landfill leachate. The initial concentration of TCE was at 0.6 mmol liter^−1^, sodium dl-lactate was at 6 mM, and methanol was at 12 mM. Lactate at 6 mM was added for the second time on day 46 in the cultures with 1 and 2 g liter^−1^ NH_4_^+^-N. The bottles were incubated at 30°C in the dark on a platform shaker set at 125 rpm.

### Microbial inoculum.

The microbial inoculum capable of TCE dehalogenation was the enrichment culture ZARA-10. ZARA-10 was developed from soil material with TCE as the chlorinated electron acceptor and lactate and methanol as the electron donors and carbon sources (14). The relative abundance of its microbial populations was previously determined using high-throughput sequencing and real-time qPCR (14). ZARA-10 inoculum contains multiple strains of *D. mccartyi* with the identified reductive dehalogenase genes *tceA*, *vcrA*, and *bvcA* and members of the *Geobacteraceae* family capable of TCE–to–*cis*-DCE dehalogenation. It also contains fermenting and homoacetogenic genera *Acetobacterium* and *Clostridium* (comprising 50% of the microbial community) and hydrogenotrophic methanogens belonging to the families *Methanobacteriales*, *Methanomicrobiales*, and *Methanococcocales* (14). Acetoclastic or mixotrophic methanogens are not present in ZARA-10 (14). The microbial composition of ZARA-10 shares many similarities with other organohalide-respiring and fermenting cultures (14, 16, 53, 54) and environmental communities from contaminated sites (15–18). Ten-milliliter culture aliquots were added to each bottle at time zero (10% [vol/vol]). For the leachate study, we also established uninoculated controls and uninoculated abiotic controls. The abiotic controls were generated by autoclaving the landfill leachate. All experimental conditions were tested in triplicate.

### Chemical analytical methods.

TCE, *cis*-DCE, VC, ethene, and methane were analyzed from 200-µl gas samples using a gas chromatograph instrument (GC-2010; Shimadzu, Columbia, MD) equipped with a flame ionization detector (FID) and an Rt-QS-Bond capillary column (Restek, Bellefonte, PA). The GC settings and analytical methods were as previously described (52). A GC equipped with a TCD was employed to measure H_2_ in the headspace of the bottles, using the methodology and conditions described by Parameswaran et al. (55). The detection limit for H_2_ was 0.32 mmol liter^−1^ (gas concentration).

Lactate, methanol, acetate, and propionate were measured via high-performance liquid chromatography (HPLC) from 1-ml liquid samples filtered through 0.2-µm syringe filters. The instrument used was a Shimadzu LC-20AT equipped with an Aminex HPX-87H column (Bio-Rad, Hercules, CA). Detection of chromatographic peaks was achieved using a photodiode array detector at 210 nm and a refractive index detector. The eluent was 2.5 mM H_2_SO_4_ and the column temperature was kept constant at 50°C. Five-point calibration curves were generated for all compounds during each run. The detection limits for organic acids and methanol were ≤0.1 mM and 0.5 mM, respectively.

Concentrations of ammonium, total nitrogen, and chemical oxygen demand were determined using Hach (Loveland, CO) analytical kits according to the manufacturer’s instructions.

### Quantitative real-time PCR methods.

DNA was extracted from triplicate pellets formed from 0.5-ml culture aliquots sampled at the beginning and end of the experiments, as previously described (53). Real-time qPCR analyses were run for the following targets: the *Dehalococcoides* 16S rRNA gene, *Geobacteraceae* 16S rRNA gene, *Archaea* 16S rRNA gene, and the FTHFS gene of homoacetogens. Triplicate reactions were set up for six-point standard curves and samples in 10-µl total volumes using 4 µl of 1/10-diluted DNA as the template. Pipetting was performed using an automated liquid handling system (epMotion 5070; Eppendorf, USA). The standard curves were produced by serially diluting 10 ng µl^−1^ plasmid DNA. The primers and probes, reagent concentrations, and thermocycler (Realplex 4S thermocycler; Eppendorf, Hauppauge, NY) conditions used were those previously published (14, 56).

### Statistical analyses.

Two-tailed parametric (Pearson) and nonparametric (Spearman) correlations were determined for reductive dehalogenation rates and ammonium concentrations. Statistical analyses were performed using IBM SS Statistic 22 software.

## References

[1] Taş N, van Eekert MH, de Vos WM, Smidt H 2010 The little bacteria that can—diversity, genomics and ecophysiology of “*Dehalococcoides*” spp. in contaminated environments. Microb Biotechnol 3:389–402. doi:10.1111/j.1751-7915.2009.00147.x. 21255338 PMC3815806

[2] Löffler FE, Yan J, Ritalahti KM, Adrian L, Edwards EA, Konstantinidis KT, Müller JA, Fullerton H, Zinder SH, Spormann AM 2013 *Dehalococcoides mccartyi* gen. nov., sp. nov., obligately organohalide-respiring anaerobic bacteria relevant to halogen cycling and bioremediation, belong to a novel bacterial class, *Dehalococcoidia* classis nov., order *Dehalococcoidales* ord. nov. and family *Dehalococcoidaceae* fam. nov., within the phylum *Chloroflexi*. Int J Syst Evol Microbiol 63:625–635. doi:10.1099/ijs.0.034926-0. 22544797

[3] Stroo H, Leeson A, Ward C 2013 Bioaugmentation for groundwater remediation, 1st ed Springer Verlag, New York, NY.

[4] Moran MJ, Zogorski JS, Squillace PJ 2007 Chlorinated solvents in groundwater of the United States. Environ Sci Technol 41:74–81. doi:10.1021/es061553y. 17265929

[5] Duhamel M, Wehr SD, Yu L, Rizvi H, Seepersad D, Dworatzek S, Cox EE, Edwards EA 2002 Comparison of anaerobic dechlorinating enrichment cultures maintained on tetrachloroethene, trichloroethene, *cis*-dichloroethene and vinyl chloride. Water Res 36:4193–4202. doi:10.1016/S0043-1354(02)00151-3. 12420924

[6] Chan WW, Grostern A, Löffler FE, Edwards EA 2011 Quantifying the effects of 1,1,1-trichloroethane and 1,1-dichloroethane on chlorinated ethene reductive dehalogenases. Environ Sci Technol 45:9693–9702. doi:10.1021/es201260n. 21955221

[7] Grostern A, Edwards EA 2006 A 1,1,1-trichloroethane-degrading anaerobic mixed microbial culture enhances biotransformation of mixtures of chlorinated ethenes and ethanes. Appl Environ Microbiol 72:7849–7856. doi:10.1128/AEM.01269-06. 17056695 PMC1694251

[8] Adamson DT, Parkin GF 2000 Impact of mixtures of chlorinated aliphatic hydrocarbons on a high-rate, tetrachloroethene-dechlorinating enrichment culture. Environ Sci Technol 34:1959–1965. doi:10.1021/es990809f.

[9] Leahy J, Shreve GS 2000 The effect of organic carbon on the sequential reductive dehalogenation of tetrachloroethylene in landfill leachates. Water Res 34:2390–2396. doi:10.1016/S0043-1354(99)00389-9.

[10] Wakida FT, Lerner DN 2005 Non-agricultural sources of groundwater nitrate: a review and case study. Water Res 39:3–16. doi:10.1016/j.watres.2004.07.026. 15607159

[11] Böhlke JK, Smith RL, Miller DN 2006 Ammonium transport and reaction in contaminated groundwater: application of isotope tracers and isotope fractionation studies. Water Resour Res 42:W05411. doi:10.1029/2005WR004349.

[12] Hong J, Tezel U, Okutman Tas D, Pavlostathis SG 2013 Influence of quaternary ammonium compounds on the microbial reductive dechlorination of pentachloroaniline. Water Res 47:6780–6789. doi:10.1016/j.watres.2013.09.014. 24075473

[13] Agency of Toxic Substances and Disease Registry. 2013 Priority list of hazardous substances. ATSDR, CDC, Atlanta, GA.

[14] Delgado AG, Kang D-W, Nelson KG, Fajardo-Williams D, Miceli JF III, Done HY, Popat SC, Krajmalnik-Brown R 2014 Selective enrichment yields robust ethene-producing dechlorinating cultures from microcosms stalled at *cis*-dichloroethene. PLoS One 9:e100654. doi:10.1371/journal.pone.0100654. 24950250 PMC4065118

[15] Sutton NB, Atashgahi S, Saccenti E, Grotenhuis T, Smidt H, Rijnaarts HH 2015 Microbial community response of an organohalide respiring enrichment culture to permanganate oxidation. PLoS One 10:e0134615. doi:10.1371/journal.pone.0134615. 26244346 PMC4526698

[16] Macbeth TW, Cummings DE, Spring S, Petzke LM, Sorenson KS 2004 Molecular characterization of a dechlorinating community resulting from in situ biostimulation in a trichloroethene-contaminated deep, fractured basalt aquifer and comparison to a derivative laboratory culture. Appl Environ Microbiol 70:7329–7341. doi:10.1128/AEM.70.12.7329-7341.2004. 15574933 PMC535138

[17] Rahm BG, Chauhan S, Holmes VF, Macbeth TW, Sorenson KS, Alvarez-Cohen L 2006 Molecular characterization of microbial populations at two sites with differing reductive dechlorination abilities. Biodegradation 17:523–534. doi:10.1007/s10532-005-9023-9. 16477354

[18] Miura T, Yamazoe A, Ito M, Ohji S, Hosoyama A, Takahata Y, Fujita N 2015 The impact of injections of different nutrients on the bacterial community and its dechlorination activity in chloroethene-contaminated groundwater. Microbes Environ 30:164–171. doi:10.1264/jsme2.ME14127. 25877696 PMC4462927

[19] Zhuang WQ, Yi S, Feng X, Zinder SH, Tang YJ, Alvarez-Cohen L 2011 Selective utilization of exogenous amino acids by *Dehalococcoides ethenogenes* strain 195 and its effects on growth and dechlorination activity. Appl Environ Microbiol 77:7797–7803. doi:10.1128/AEM.05676-11. 21890673 PMC3209135

[20] He J, Holmes VF, Lee PK, Alvarez-Cohen L 2007 Influence of vitamin B_12_ and cocucultures on the growth of *Dehalococcoides* isolates in define*d* medium. Appl Environ Microbiol 73:2847–2853. doi:10.1128/AEM.02574-06. 17337553 PMC1892872

[21] Zhuang W-Q, Yi S, Bill M, Brisson VL, Feng X, Men Y, Conrad ME, Tang YJ, Alvarez-Cohen L 2014 Incomplete Wood-Ljungdahl pathway facilitates one-carbon metabolism in organohalide-respiring *Dehalococcoides mccartyi*. Proc Natl Acad Sci USA 111:6419–6424. doi:10.1073/pnas.1321542111. 24733917 PMC4035967

[22] McLean JE, Ervin J, Zhou J, Sorensen DL, Dupont RR 2015 Biostimulation and bioaugmentation to enhance reductive dechlorination of TCE in a long-term flow through column study. Ground Water Monit Remediat 35:76–88. doi:10.1111/gwmr.12113.

[23] Meckenstock RU, Elsner M, Griebler C, Lueders T, Stumpp C, Aamand J, Agathos SN, Albrechtsen H-J, Bastiaens L, Bjerg PL, Boon N, Dejonghe W, Huang WE, Schmidt SI, Smolders E, Sørensen SR, Springael D, van Breukelen BM 2015 Biodegradation: updating the concepts of control for microbial cleanup in contaminated aquifers. Environ Sci Technol 49:7073–7081. doi:10.1021/acs.est.5b00715. 26000605

[24] Lee PK, He J, Zinder SH, Alvarez-Cohen L 2009 Evidence for nitrogen fixation by “*Dehalococcoides ethenogenes*” strain 195. Appl Environ Microbiol 75:7551–7555. doi:10.1128/AEM.01886-09. 19820162 PMC2786412

[25] Lay J, Li Y, Noike T 1998 The influence of pH and ammonia concentration on the methane production in high-solids digestion processes. Water Environ Res 70:1075–1082. doi:10.2175/106143098X123426.

[26] Müller T, Walter B, Wirtz A, Burkovski A 2006 Ammonium toxicity in bacteria. Curr Microbiol 52:400–406. doi:10.1007/s00284-005-0370-x. 16604417

[27] Kadam PC, Boone DR 1996 Influence of pH on ammonia accumulation and toxicity in halophilic, methylotrophic methanogens. Appl Environ Microbiol 62:4486–4492. PubMed. 16535465 10.1128/aem.62.12.4486-4492.1996 PMC1389003

[28] Sprott GD, Patel GB 1986 Ammonia toxicity in pure cultures of methanogenic bacteria. Syst Appl Microbiol 7:358–363. doi:10.1016/S0723-2020(86)80034-0.

[29] Hajarnis SR, Ranade DR 1994 Revival of *Methanobacterium formicicum* after its inhibition by high concentrations of ammonia. Lett Appl Microbiol 18:254–256. doi:10.1111/j.1472-765X.1994.tb00862.x.

[30] Garcia ML, Angenent LT 2009 Interaction between temperature and ammonia in mesophilic digesters for animal waste treatment. Water Res 43:2373–2382. doi:10.1016/j.watres.2009.02.036. 19321185

[31] Kjeldsen P, Barlaz MA, Rooker AP, Baun A, Ledin A, Christensen TH 2002 Present and long-term composition of MSW landfill leachate: a review. Crit Rev Environ Sci Technol 32:297–336. doi:10.1080/10643380290813462.

[32] Rajagopal R, Massé DI, Singh G 2013 A critical review on inhibition of anaerobic digestion process by excess ammonia. Bioresour Technol 143:632–641. doi:10.1016/j.biortech.2013.06.030. 23835276

[33] Mahmoud M, Parameswaran P, Torres CI, Rittmann BE 2014 Fermentation pre-treatment of landfill leachate for enhanced electron recovery in a microbial electrolysis cell. Bioresour Technol 151:151–158. doi:10.1016/j.biortech.2013.10.053. 24231265

[34] Salerno MB, Park W, Zuo Y, Logan BE 2006 Inhibition of biohydrogen production by ammonia. Water Res 40:1167–1172. doi:10.1016/j.watres.2006.01.024. 16513155

[35] Gallert C, Winter J 1997 Mesophilic and thermophilic anaerobic digestion of source-sorted organic wastes: effect of ammonia on glucose degradation and methane production. Appl Microbiol Biotechnol 48:405–410. doi:10.1007/s002530051071.

[36] Sawayama S, Tada C, Tsukahara K, Yagishita T 2004 Effect of ammonium addition on methanogenic community in a fluidized bed anaerobic digestion. J Biosci Bioeng 97:65–70. doi:10.1016/S1389-1723(04)70167-X. 16233591

[37] Sossa K, Alarcón M, Aspé E, Urrutia H 2004 Effect of ammonia on the methanogenic activity of methylaminotrophic methane producing *Archaea* enriched biofilm. Anaerobe 10:13–18. doi:10.1016/j.anaerobe.2003.10.004. 16701495

[38] Christensen TH, Kjeldsen P, Bjerg PL, Jensen DL, Christensen JB, Baun A, Albrechtsen H, Heron G 2001 Biogeochemistry of landfill leachate plumes. Appl Geochem 16:659–718. doi:10.1016/S0883-2927(00)00082-2.

[39] Arigala SG, Tsotsis TT, Webster IA, Yortsos YC, Kattapuram JJ 1995 Gas generation, transport, and extraction in landfills. J Environ Eng 121:33–44. doi:10.1061/(ASCE)0733-9372(1995)121:1(33).

[40] Chang YC, Jung K, Yoo YS 2003 Anaerobic degradation of *cis*-1,2-dichloroethylene by cultures enriched from a landfill leachate sediment. J Microbiol Biotechnol 13:366–372.

[41] Fennell DE, Gossett JM 1998 Modeling the production of and competition for hydrogen in a dechlorinating culture. Environ Sci Technol 32:2450–2460. doi:10.1021/es980136l.

[42] He J, Sung Y, Dollhopf ME, Fathepure BZ, Tiedje JM, Löffler FE 2002 Acetate versus hydrogen as direct electron donors to stimulate the microbial reductive dechlorination process at chloroethene-contaminated sites. Environ Sci Technol 36:3945–3952. doi:10.1021/es025528d. 12269747

[43] Heimann AC, Friis AK, Jakobsen R 2005 Effects of sulfate on anaerobic chloroethene degradation by an enriched culture under transient and steady-state hydrogen supply. Water Res 39:3579–3586. doi:10.1016/j.watres.2005.06.029. 16085242

[44] Men Y, Seth EC, Yi S, Allen RH, Taga ME, Alvarez-Cohen L 2014 Sustainable growth of *Dehalococcoides mccartyi* 195 by corrinoid salvaging and remodeling in defined lactate-fermenting consortia. Appl Environ Microbiol 80:2133–2141. doi:10.1128/AEM.03477-13. 24463969 PMC3993147

[45] Fennell DE, Gossett JM, Zinder SH 1997 Comparison of butyric acid, ethanol, lactic acid, and propionic acid as hydrogen donors for the reductive dechlorination of tetrachloroethene. Environ Sci Technol 31:918–926. doi:10.1021/es960756r.

[46] Booker R, Pavlostathis SG 2000 Microbial reductive dechlorination of hexachloro-1,3-butadiene in a methanogenic enrichment culture. Water Res 34:4437–4445. doi:10.1016/S0043-1354(00)00214-1.

[47] Aulenta F, Gossett JM, Papini MP, Rossetti S, Majone M 2005 Comparative study of methanol, butyrate, and hydrogen as electron donors for long-term dechlorination of tetrachloroethene in mixed anerobic cultures. Biotechnol Bioeng 91:743–753. doi:10.1002/bit.20569. 16007584

[48] Poggivaraldo HM, Tingley J, Oleszkiewicz JA 1991 Inhibition of growth and acetate uptake by ammonia in batch anaerobic digestion. J Chem Technol Biotechnol 52:135–143.

[49] Braun R, Huber P, Meyrath J 1981 Ammonia toxicity in liquid piggery manure digestion. Biotechnol Lett 3:159–164. doi:10.1007/BF00239655.

[50] Hansen KH, Angelidaki I, Ahring BK 1998 Anaerobic digestion of swine manure: inhibition by ammonia. Water Res 32:5–12. doi:10.1016/S0043-1354(97)00201-7.

[51] Angenent LT, Sung S, Raskin L 2002 Methanogenic population dynamics during startup of a full-scale anaerobic sequencing batch reactor treating swine waste. Water Res 36:4648–4654. doi:10.1016/S0043-1354(02)00199-9. 12418668

[52] Delgado AG, Parameswaran P, Fajardo-Williams D, Halden RU, Krajmalnik-Brown R 2012 Role of bicarbonate as a pH buffer and electron sink in microbial dechlorination of chloroethenes. Microb Cell Fact 11:128. doi:10.1186/1475-2859-11-128. 22974059 PMC3511292

[53] Ziv-El M, Delgado AG, Yao Y, Kang DW, Nelson KG, Halden RU, Krajmalnik-Brown R 2011 Development and characterization of DehaloR^2, a novel anaerobic microbial consortium performing rapid dechlorination of TCE to ethene. Appl Microbiol Biotechnol 92:1063–1071. doi:10.1007/s00253-011-3388-y. 21667274

[54] Men Y, Lee PK, Harding KC, Alvarez-Cohen L 2013 Characterization of four TCE-dechlorinating microbial enrichments grown with different cobalamin stress and methanogenic conditions. Appl Microbiol Biotechnol 97:6439–6450. doi:10.1007/s00253-013-4896-8. 23640361 PMC6436544

[55] Parameswaran P, Torres CI, Lee HS, Rittmann BE, Krajmalnik-Brown R 2011 Hydrogen consumption in microbial electrochemical systems (MXCs): the role of homo-acetogenic bacteria. Bioresour Technol 102:263–271. doi:10.1016/j.biortech.2010.03.133. 20430615

[56] Delgado AG, Fajardo-Williams D, Popat SC, Torres CI, Krajmalnik-Brown R 2014 Successful operation of continuous reactors at short retention times results in high-density, fast-rate *Dehalococcoides* dechlorinating cultures. Appl Microbiol Biotechnol 98:2729–2737. doi:10.1007/s00253-013-5263-5. 24085396

