# Supplementary figures and images for: Impact of Ammonium on Syntrophic Organohalide-Respiring and Fermenting Microbial Communities
Source: mSphere. 2016 Apr 20;1(2):e00053-16. doi: 10.1128/mSphere.00053-16 (PMC4894693; doi:10.1128/mSphere.00053-16)

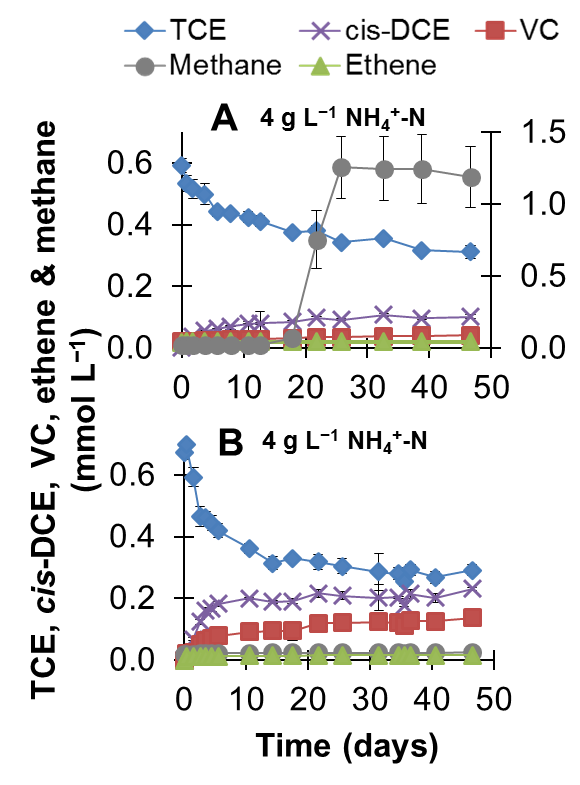


**Figure S1**

Supplement: Figure S1 [file sph002162062sf2.docx]

**
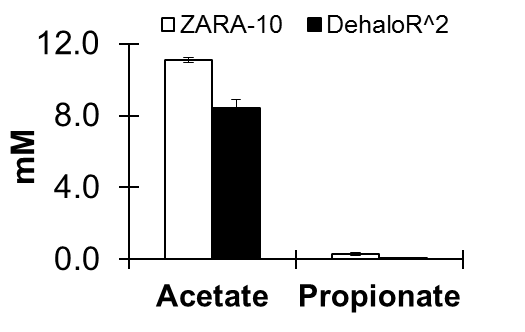
**

**Figure S2**

Supplement: Figure S2 [file sph002162062sf3.docx]

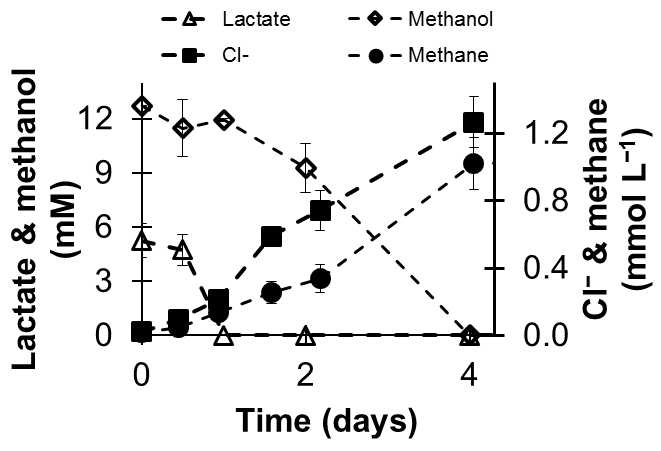


**Figure S3**

Supplement: Figure S3 [file sph002162062sf4.docx]
