# Supplementary material for: Impact of Ammonium on Syntrophic Organohalide-Respiring and Fermenting Microbial Communities
Source: mSphere. 2016 Apr 20;1(2):e00053-16. doi: 10.1128/mSphere.00053-16 (PMC4894693; doi:10.1128/mSphere.00053-16)
Supplement: Text S1 [file sph002162062s1.docx]

**SUPPLEMENTAL MATERIAL**

**Effect of 4 g L^−1^ NH_4_^+^-N on reductive dehalogenation, methanogenesis and fermentation**

An additional experiment was setup using a concentration of 4 g L^−1^ NH_4_^+^-N as described in the Materials and Methods in the manuscript. The purpose of the experiment was to verify the following:

- Production of dehalogenation by-products at a higher concentration of ammonium
- Effect of 4 g L^−1^ NH_4_^+^-N on methane production and fermentation of lactate to acetate and propionate or mainly acetate
- Response of another organohalide-respiring culture to these conditions

The additional microbial inoculum tested in these experiments was DehaloR^2. DehaloR^2 was developed from a contaminated sediment and has been thoroughly characterized with respect to its microbial composition (1-3). ZARA-10 and DehaloR^2 are similar in terms of organohalide-respiring and fermenting populations, except methanogenesis is suppressed in DehaloR^2.

**References**

1. **Ziv-El M, Delgado AG, Yao Y, Kang DW, Nelson KG, Halden RU, Krajmalnik-Brown R.** 2011. Development and characterization of DehaloR^2, a novel anaerobic microbial consortium performing rapid dechlorination of TCE to ethene. Applied Microbiology and Biotechnology **92:**1063-1071.

2. **Delgado AG, Parameswaran P, Fajardo-Williams D, Halden RU, Krajmalnik-Brown R.** 2012. Role of bicarbonate as a pH buffer and electron sink in microbial dechlorination of chloroethenes. Microbial Cell Factories **11**.

3. **Delgado AG, Fajardo-Williams D, Popat SC, Torres CI, Krajmalnik-Brown R.** 2014. Successful operation of continuous reactors at short retention times results in high-density, fast-rate *Dehalococcoides* dechlorinating cultures. Applied Microbiology and Biotechnology **98:**2729-2737.
